# Taxonomical over splitting in the *Rhodnius prolixus* (Insecta: Hemiptera: Reduviidae) clade: Are *R*. *taquarussuensis* (da Rosa et al., 2017) and *R*. *neglectus* (Lent, 1954) the same species?

**DOI:** 10.1371/journal.pone.0211285

**Published:** 2019-02-07

**Authors:** Juliana Damieli Nascimento, João Aristeu da Rosa, Fabian C. Salgado-Roa, Carolina Hernández, Carolina Pardo-Diaz, Kaio Cesar Chaboli Alevi, Amanda Ravazi, Jader de Oliveira, Maria Tercília Vilela de Azeredo Oliveira, Camilo Salazar, Juan David Ramírez

**Affiliations:** 1 Instituto de Biologia, Universidade Estadual de Campinas (UNICAMP), Campinas, SP, Brasil; 2 Laboratório de Parasitologia, Departamento de Ciências Biológicas, Faculdade de Ciências Farmacêuticas, Universidade Estadual Paulista “Júlio de Mesquita Filho” (UNESP), Araraquara, SP, Brasil; 3 Grupo de Genética Evolutiva, Filogeografía y Ecología de Biodiversidad Neotropical, Programa de Biología, Facultad de Ciencias Naturales y Matemáticas, Universidad del Rosario, Bogotá, Colombia; 4 Departamento de Ciencias Biológicas, Universidad de los Andes, Bogotá, Colombia; 5 Grupo de Investigaciones Microbiológicas-UR (GIMUR), Programa de Biología, Facultad de Ciencias Naturales y Matemáticas, Universidad del Rosario, Bogotá, Colombia; 6 Laboratório de Biologia Celular, Departamento de Biologia, Instituto de Biociências, Letras e Ciências Exatas, Universidade Estadual Paulista “Júlio de Mesquita Filho” (UNESP), São José do Rio Preto, SP, Brasil; Onderstepoort Veterinary Institute, SOUTH AFRICA

## Abstract

The use of subtle features as species diagnostic traits in taxa with high morphological similarity sometimes fails in discriminating intraspecific variation from interspecific differences, leading to an incorrect species delimitation. A clear assessment of species boundaries is particularly relevant in disease vector organisms in order to understand epidemiological and evolutionary processes that affect transmission capacity. Here, we assess the validity of the recently described *Rhodnius taquarussuensis* (da Rosa et al., 2017) using interspecific crosses and molecular markers. We did not detect differences in hatching rates in interspecific crosses between *R*. *taquarussuensis* and *R*. *neglectus* (Lent, 1954). Furthermore, genetic divergence and species delimitation analyses show that *R*. *taquarussuensis* is not an independent lineage in the *R*. *prolixus* group. These results suggest that *R*. *taquarussuensis* is a phenotypic form of *R*. *neglectus* instead of a distinct species. We would like to stress that different sources of evidence are needed to correctly delimit species. We consider this is an important step in understanding vectorial Chagas disease spread and transmission.

## Introduction

The study of the speciation process requires a complete understanding of the phenotypic variation present across the range of the study taxa. This is particularly challenging in organisms where morphological differences are subtle or not obvious, and where other aspects of their biology such as reproduction, ecology, phenology and life traits are also unknown. An increasing number of studies have documented “cryptic” speciation throughout the tree of life (i.e. taxa that cannot readily be distinguished morphologically, yet evidence indicates they are on different evolutionary trajectories). However, such descriptions have been done in absence of a clear definition of what a cryptic species is, and often using alpha taxonomy as the sole approach for detecting and classifying new species [1–4]. This can lead to false species diagnosis when unreliable traits (those lacking discontinuous, nonoverlapping patterns of variation) are used [5], which is particularly important when delimiting vector species with medical relevance, as this directly impacts the control of the diseases transmitted by them.

The subfamily Triatominae has 18 genera, with *Panstrongylus* (Berg, 1879), *Rhodnius* (Stål, 1859) and *Triatoma* (Laporte, 1832) being the most epidemiologically important genera, since they are the main species responsible for the transmission of *Trypanosoma cruzi* (Chagas, 1909), the etiologic agent of Chagas disease [6, 7]. The identification of these three genera is straightforward and is based on the insertion of the antennae on the head, which is macroscopically perceptible: in *Panstrongylus* the antennae are inserted near the eyes, in *Rhodnius* these appendages are on the anterior portion of the head, and in *Triatoma* they are located on the middle portion of the head [8, 9]. Nonetheless, the most recent Triatominae phylogeny showed that the only monophyletic genus is *Rhodnius* [9–11]. Also, species delimitation within these genera remains problematic [12]. In particular, species of *Rhodnius* show low morphological variation and their complex identification relies on few morphological traits and/or mtDNA divergence [11, 13–16]. For example, it is difficult to differentiate between *R*. *neglectus* and *R*. *prolixus* (Stål, 1859) [17], *R*. *robustus* (Larrousse, 1827) and *R*. *montenegrensis* (da Rosa et al., 2012) [18], *R*. *amazonicus* (Almeida, Santos and Sposina, 1973) and *R*. *pictipes* (Stål, 1872) [19], *R*. *pictipes* and *R*. *stali* (Lent, Jurberg and Galvão) [20], among many other examples.

Moreover, the classic division of *Rhodnius* presents additional challenges. The genus is divided into three groups: *prolixus*, *pictipes* and *pallescens*. The first two are found east of the Andes (*cis*-Andean), while the third is distributed west of the Andes (*trans*-Andean) [21–23]. The phylogenetic relationships among these groups are still under debate, especially the position of the *pictipes* group that was initially considered closer to the *pallescens* group, but recent evidence found it as sister to the *prolixus* group [23–26].

Because *Rhodnius* has an intrinsic relation with the propagation of *T*. *cruzi* and *T*. *rangeli* (Tejera, 1920), resolving its phylogenetic relationships and accurately differentiating its species is a first step to determine the epidemiological threat associated to each species, as well as to understand their ecology and population dynamics [8, 23, 27].

Recently, a new species of the genus *Rhodnius*, *R*. *taquarussuensis*, was described based on phenotypic and cytogenetic traits [22]. This is the only species of the *prolixus* group that has dispersed heterochromatin throughout the nucleus and autosomes, and it is morphologically similar to *R*. *neglectus* [22, 28]. However, the specific status of *R*. *taquarussuensis* requires a more rigorous confirmation that implements both genetic data and tests of reproductive isolation. Here, we used six molecular markers and performed crosses between *R*. *taquarussuensis* and *R*. *neglectus* in order to address whether the former is a valid species.

## Methods

### Sampling and DNA extraction

Individuals of *R*. *taquarussuensis* were collected in Taquarussu, Mato Grosso do Sul, Brazil (-22.48 Lat, -53.35 Long; Table 1) and those of *R*. *neglectus* were collected in Formoso, Goiás, Brazil (-13.65 Lat, -48.88 Long; Table 1) and maintained in the Triatominae insectary of the School of Pharmaceutical Sciences, São Paulo State University (UNESP), Araraquara, São Paulo, Brazil. *Rhodnius prolixus* were collected in Arauca (7.08 Lat, -70.75 Long), Fortul (6.78 Lat, -71.76 Long), Puerto Rondón (6.28 Lat, -71.10 Long) and Saravena (6.95 Lat, -71.87 Long) in Colombia (Table 1). UNIVERSIDAD DEL ROSARIO provided the field permit from ANLA (Autoridad Nacional de Licensias ambientales) 63257–2014. DNA was extracted from the head, legs and intestine using the DNeasy Blood & Tissue Kit (Qiagen), following the manufacturer’s protocol. The DNA concentration was determined using a NanoDrop 1000 Spectrophotometer V3.7 (Thermo Fisher Scientific, Wilmington, DE, USA) and stored at −20°C.

**Table 1:** Genes, primer information and accession numbers.

Symbol	Gene name	*Rn*	*Rp*	*Rt*	Primers (5'-3')	Tm (°C)	Fragment size (pb)	Accession numbers
CYTB	Cytochrome b**	6	5	8	R: GCW CCA ATT CAR GTT ART AA F: GGA CGW GGW ATT TAT TAT GGA TC	50	659	MH704746—MH704764
ND4	NADH dehydrogenase 4**	5	5	15	F: TAA TTC GTT GTC ATG GTA ATG F: TCA ACA TGA GCC CTT GGA AG	53	560	MH704765—MH704779
PCB	Putative chitin binding peritrophin-a domain protein	8	5	5	R: CAC TAC GGG TCG TGA AGG TT F: ACA TCC TTG GCC ACA AGA AC	55	757	MH704780—MH704797
TOPO	DNA topoisomerase	5	6	5	F: CAA CAC TTG TAA CCC GAG CA F: ATC ATT GGC CGC ATC TTT AG	56	604	MH704798—MH704813
URO	Uroporphyrinogen decarboxylase	11	6	6	R: TTA AGG GCA GCA AGA GGA GA F: AAC ACA TTT CCT GGC CAA AG	54	563	MH704814—MH704828
ZNFP	Toll-like-2. Transmembrane receptor with TIR domain binding	5	5	5	F: TCC TTG CGG TAA TGA TGT GA F: CTC GAA TGG TGT ACG TGG TG	54	588	MH704829—MH704852

Gene IDs correspond to those in the *Rhodnius* genome GFF file annotation.

**Published before. *Rn*: *R*. *neglectus; Rp*: *R*. *prolixus; Rt*: *R*. *taquarussuensis*

### Loci amplification and sequencing

We amplified and sequenced two mitochondrial gene fragments, Cytochrome b (CYTB) and Mitochondrially Encoded NADH Dehydrogenase 4 (ND4) using the conditions reported elsewhere [29]. We also designed primers to develop new coding nuclear markers in *Rhodnius*. In order to do this, we used the *R*. *prolixus* genome available in VectorBase (https://www.vectorbase.org/organisms/rhodnius-prolixus) and, from the GFF file, we selected four large exon markers (≥700 bp) using a custom script. We then used BLASTn to compare these exons to the *R*. *prolixus* transcriptome and thus confirm they were single copy markers. Then, we verified the identity of the selected exons in Uniprot with the ID codes registered in the genome. Finally, we designed primers for these loci using Primer 3 [30]. The resulting nuclear markers are Putative chitin binding peritrophin-a (PCB), DNA topoisomerase (TOPO), Uroporphyrinogen decarboxylase (URO) and Toll-Like-2. Transmembrane receptor with TIR domain binding (ZNFP) (Table 1 and Table 2).

**Table 2:** Nuclear markers (single copy exons) designed in this study.

Gen	Annotation in the *R*. *prolixus* genome							Region amplified		
	Gene ID		Scaffold	Strand	Start	End	Size (bp)	Location	Start	End
ZNFP	RPRC009262-RA	Tl-like-2: Toll-like-2. Transmembrane receptor with TIR domain binding	KQ034161	+	481476	486977	5501	Exon 1	481599	482146
URO	RPRC013534-RA	UROD: Uroporphyrinogen decarboxylase	KQ034105	-	970351	971418	1067	Exon 1	970699	971261
TOPO	RPRC012703-RA	DNA topoisomerase	KQ034259	+	391034	406927	15893	Exon 3	404730	405333
PCB	RPRC001863-RA	Putative chitin binding peritrophin-a	KQ034056	+	8334541	8342490	7949	Exon 3	8335296	8336052

Gene IDs correspond to those in the *Rhodnius* genome GFF file annotation.

PCR reactions had a final volume of 25 μl, consisting of 12.5 μl of GoTaq Green Master Mix (Promega, Madison, WI, USA), 1.25 μL (10 μM) of each primer and, 5.0 μl of DNA (20 ng) and 5μL of H_2_O. Amplification was conducted in a Thermal Cycler 4000 (Bio-Rad La-boratories, Inc., Hercules, CA, USA). The following PCR cycling conditions were used: 94°C for 5 min; 40 cycles of 94°C for 1 min, 50–56°C for 1 min (Table 1), and a final extension at 72°C for 10 min. PCR success was verified by electrophoresis on agarose gel stained with Fast SYBR Green (Applied Biosystems, Foster City, CA, USA) and a molecular weight marker (Promega) adding 2μl of each PCR product. The samples were purified using the PCR kit ExoSAP-IT Product Cleanup (Affymetrix, Santa Clara, CA, USA) and sequenced at Macrogen Inc. (Seoul, Korea).

### Sequence analyses

Gene sequences were read, edited and aligned with CLC Main Workbench (Qiagen). For nuclear loci, haplotype inference for heterozygous calls was conducted using the PHASE algorithm implemented in DnaSP v5 [31], accepting haplotypes with a confidence higher than 90% after running 5,000 interactions per simulation. Then, we created alignments for each locus using MUSCLE [32] with the default parameters. These alignments were visualized and corrected by hand in MEGA X [33]. Finally, we translated the sequences to proteins in order to verify for stop codons using MESQUITE 3.04 [34].

### Molecular phylogenetics and species delimitation

In order to assess the position of *R*. *taquarussuensis* within the group *prolixus*, we downloaded from the Genbank all CYTB sequences available for this group and one from *Triatoma infestans* (outgroup; S1 Table) using the following Entrez ⁠line: “esearch -db nucleotide -query "<organism> CYTB" | efetch -format fasta” [35]. We combined these data with our sequences and estimated a phylogenetic tree for the group *prolixus* using a Maximum likelihood (ML) optimization in IQ-TREE [36]⁠. The substitution model for CYTB was established in the same software, selecting the model with the lowest BIC score. Node support was calculated with 1,000 ultrafast bootstrap replicates.

We also explored the phylogenetic relationships between *R*. *prolixus*, *R*. *neglectus* and *R*. *taquarussuensis*, concatenating all loci (nuclear and mitochondrial; 3731 bp long alignment) in Mesquite 3.04 [34] ⁠and estimating a ML phylogenetic tree with in IQ-TREE [36]⁠. We allowed each locus to have its own substitution model, and node support was accessed as above. We also conducted a Bayesian analysis independently for each locus using BEAST 2.5, implementing linked and unlinked tree models [37]. We inferred the nucleotide substitution model, range of the rate of heterogeneity, and proportion of invariant positions during the MCMC analysis with the bModelTest package [38], with transition-transversion split option and empirical frequencies. We ran 10’000,000 generations sampling every 1,000 generations and used TRACER [39] to confirm the coverage of the chain (i.e. effective sample size >200). TreeAnnotator [37] was used to construct a consensus tree per locus and the initial 10% trees were discarded as burn-in. We superimposed and plotted consensus gene trees constructing a Multiphylo object with the densiTree function in R [40].

As the resulting ML and Bayesian topologies were identical, we used the ML tree as input for a species delimitation analysis intended to determine the species boundaries between *R*. *taquarussuensis*, *R*. *neglectus* and *R*. *prolixus*. This analysis was carried out under a phylogenetic species concept using the Bayesian and ML version of PTP with 500,000 MCMC generations, thinning = 100 and burn-in = 0.1 [41]⁠. PTP implements a non-ultrametric phylogeny to model speciation rate as the number of substitutions reflected as branch lengths, assuming that the number of substitutions between species are significantly higher than the number of substitutions within species.

### Genetic differentiation analysis and haplotype networks

We calculated segregating sites (SS), nucleotide diversity (π), haplotype diversity (Hd), number of synonymous and non-synonymous substitutions, singletons and Tajima’s D with DnaSP v5 [31]⁠. We did not calculate relative genetic differentiation (F_ST_) as it has been shown to be overestimated when low nucleotide diversities are obtained [42], as in our dataset (Table 3). Instead, we calculated an absolute divergence measure (D_XY_) and its nucleotide diversity corrected version (Da) with DnaSP v5. D_XY_ was visualized as a heatmap drawn with the R package *“fields”*. We also calculated Kimura 2 parameter distance (K2P) which has been previously used in triatomines to validate different species [43].

**Table 3:** Summary statistics for each locus.

Species	Gene	Pi (π)	SS	Tajima's D*	Hd	Synonymous sites	Non- synonymous sites	Singletons
*R*. *neglectus*	CYTB	0	0	0	0	0	0	0
	ND4	0.00089	1	-0.61	0.5	0	1	1
	PCB	0.0012	2	1.085	0.49	1	1	0
	TOPO	0	0	0	0	0	0	0
	URO	0.00015	1	-1.15	0.083	0	1	1
	ZNFP	0	0	0	0	0	0	0
*R*. *taquarussuensis*	CYTB	1.00E-07	1	-1.05	0.25	1	0	1
	ND4	0	0	0	0	0	0	0
	PCB	0.00074	1	1.38	0.53	0	1	0
	TOPO	0.00353	6	0.02	0.62	3	3	0
	URO	0.00143	4	-1.38	0.56	0	4	3
	ZNFP	0.00091	1	0.85	0.81	0	1	0
*R*. *prolixus*	CYTB	0.00965	13	-1.1	1	2	12	12
	ND4	0.00714	4	0	1	1	3	4
	PCB	0.00141	3	0.021	0.35	1	2	0
	TOPO	0.00028	1	-1.14	0.17	1	0	1
	URO	0.00328	4	1.39	0.77	1	3	0
	ZNFP	0.00181	2	1.031	0.53	0	2	0

*None of the Tajima’s D were significant.

Genetic clustering between *R*. *neglectus* and *R*. *taquarussuensis* was validated with a discriminant analysis of principal components (DAPC) performed with both nDNA and mtDNA using the ‘*adegenet*’ R package [44]. We did this by transforming fasta sequences into a genind object that contains individual genotypes and loading it into ‘*adegenet*’ [44]⁠. We performed a principal component analysis (PCA) on these data and retained the first two components (that accounted for >90% of the total variation in both mtDNA and nDNA). We then applied a discriminant analysis using the dapc function and assuming two prior groups (i.e. two species). This produced a single canonical function that summarizes the individual genetic variability, which was then visualized with a density plot. Finally, we constructed haplotype median-joining networks per locus with POPART [45].

### Interspecific crosses

As a first attempt to determine the presence of reproductive isolation between *R*. *taquarussuensis* and *R*. *neglectus*, we performed interspecific (direct and reciprocal) and conspecific crosses. These were conducted in the Triatominae insectary of the School of Pharmaceutical Sciences, São Paulo State University (UNESP), Araraquara, São Paulo, Brazil, following the methodology established by Costa et al. [46] and Mendonça et al. [47]. Each cross was replicated three times for a total of 12 matings. First, insects were sexed as 5th instar nymphs [48], and males and females were kept separately until they reached the adult stage [49]. Then, a virgin female was placed with a male inside a plastic box (5cm diameter × 10cm height) for a maximum period of 120 days and kept at room temperature. The success or failure of mating was recorded by direct observation. After seven days, we collected the eggs of each cross weekly throughout the females’ oviposition period (120 days). The eggs collected were placed inside a plastic box (5cm diameter × 10 cm height) and their hatching was recorded weekly.

We calculated hatching success of the interspecific crosses as a measure of egg viability relative to conspecific crosses. A likelihood approximation was implemented in Betabino 1.1 [50] to analyze these data. Because using a binomial model alone does not account for the variation in hatching rate among families in each type of cross, Betabino fits a beta-binomial distribution to count data (in our case, number of eggs that hatched), thus solving this issue. Four alternative models that contrast the number of parameters in the data (i.e. mean and variance in the hatching rate) were tested. For details see http://www.ucl.ac.uk/~ucbhdjm/bin/betabino/betabino.pdf and the appendix section in [50].

## Results

### Molecular phylogenetics and species delimitation

**Fig 1:**
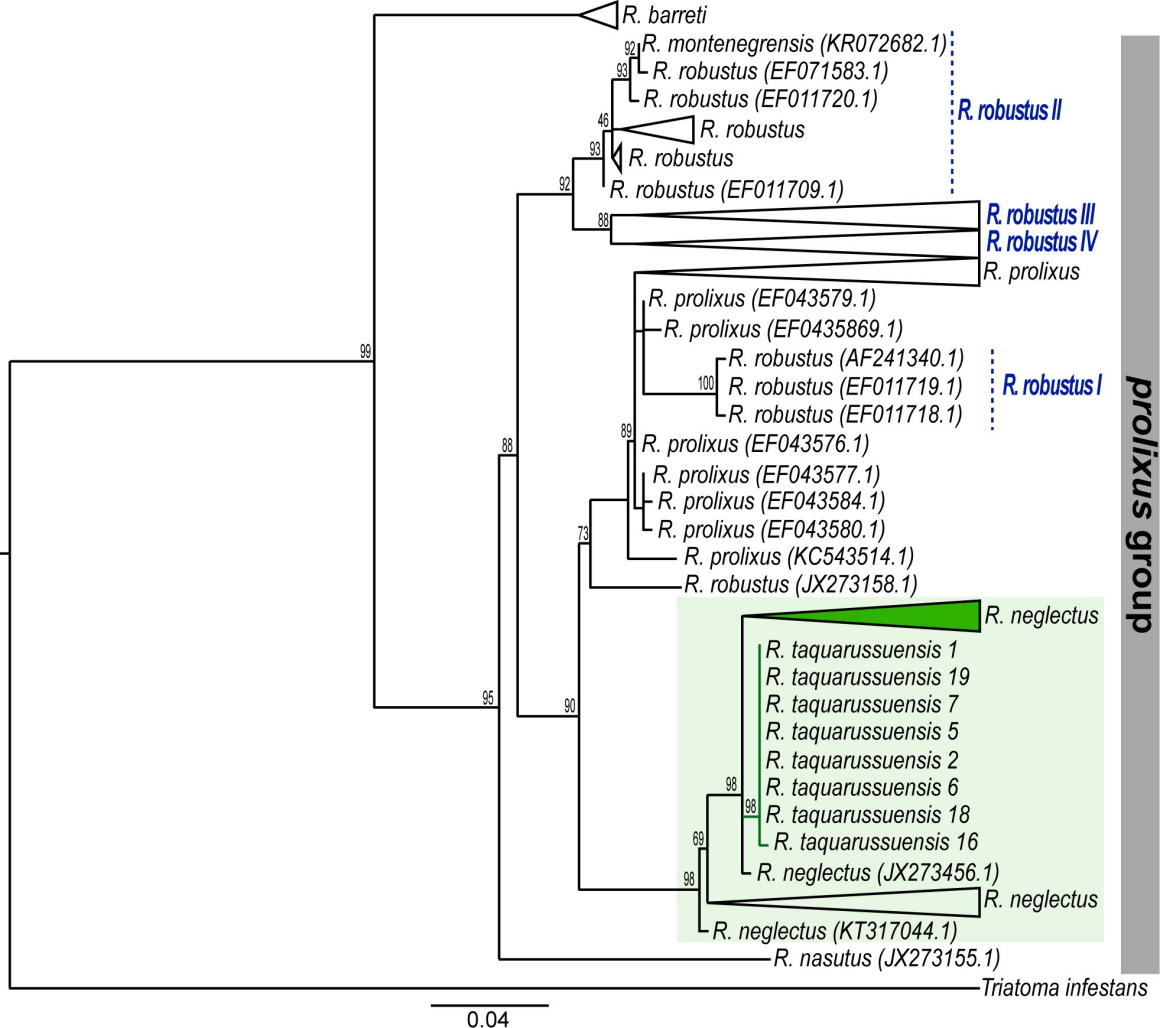
Maximum Likelihood tree for *Rhodnius* based on CYTB. Numbers on the nodes are bootstrap supports. The vertical bar on the right highlight the *prolixus* group. The focal species, namely, *R*. *taquarussuensis* and *R*. *neglectus*, are highlighted in the green square. Green branches and the collapsed clade (green triangle) correspond to the sequences obtained here for *R*. *taquarussuensis* and *R*. *neglectus* respectively.

All sequences obtained for this study were deposited in the Genbank and their accession numbers are found in Table 1. Our dataset for the CYTB gene consisted of 162 sequences corresponding to six species and confirmed the phylogenetic relationships previously shown by Monteiro et al. [11]. Briefly, the ML topology obtained with this gene (evolution model TN+F+I; BIC score 4339.957) revealed that the *prolixus* group is subdivided into two clades, one exclusively formed by *R*. *barreti* (Abad-Franch, Palomeque and Monteiro, 2013), and the second consisting of *R*. *robustus*, *R*. *montenegrensis*, *R*. *prolixus*, *R*. *neglectus*, *R*. *nasutus* (Stål, 1859), and *R*. *taquarussuensis*. The relations within this latter clade are complicated. For example, we recovered the four groups previously described for *R*. *robustus* [11], where *R*. *robustus-*I falls inside the *R*. *prolixus* clade, and *R*. *montenegrensis* is part of *R*. *robustus-*II (Fig 1 and S1 Fig). Additionally, the species *R*. *neglectus* is recovered as sister to *R*. *prolixus* and contains all individuals from the newly described species *R*. *taquarussuensis*, which although monophyletic, has virtually no differentiation from *R*. *neglectus* (Fig 1 and S1 Fig).

**Fig 2:**
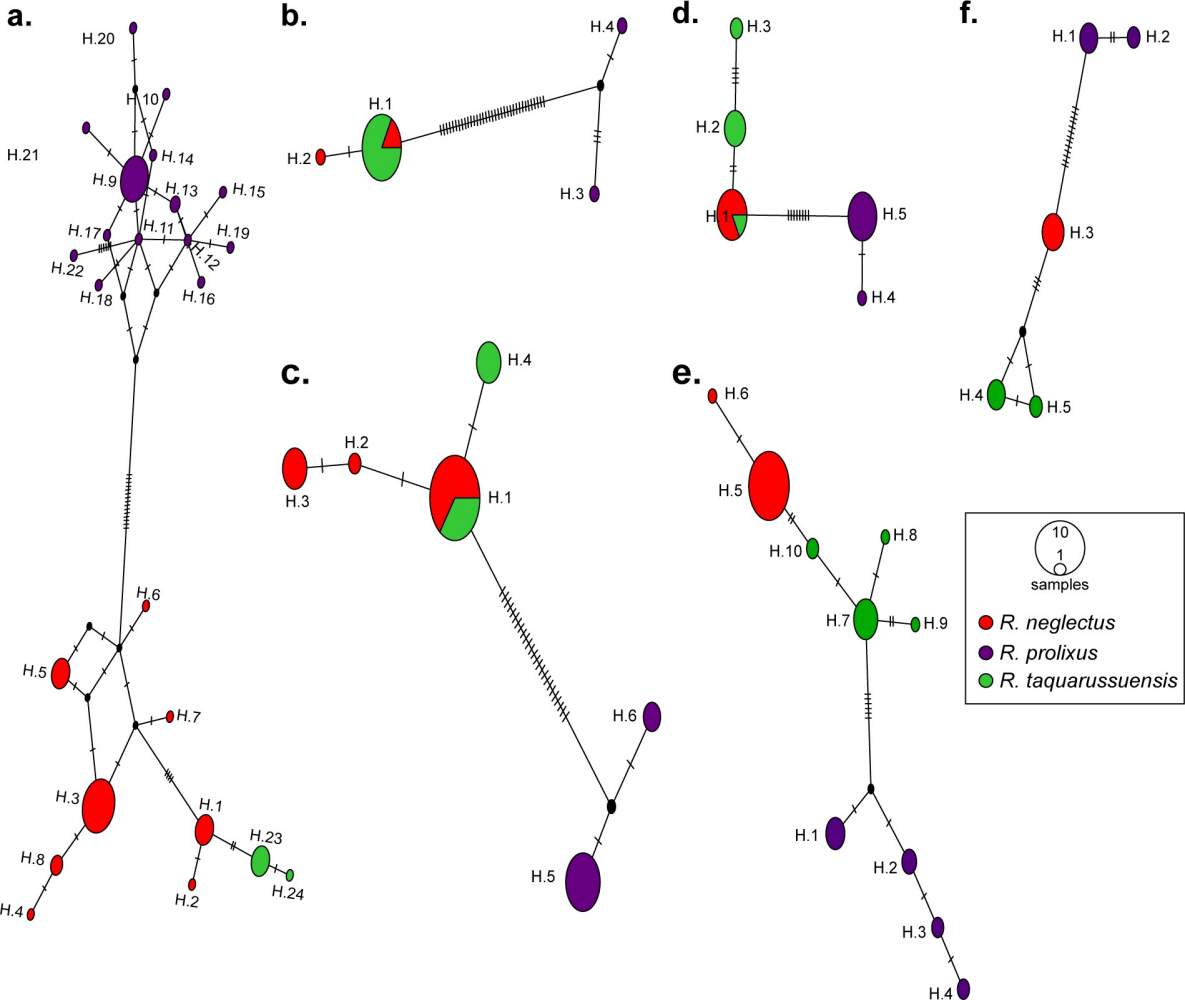
Haplotype networks. (a) CYTB; (b) ND4; (c) PCB; (d) TOPO; (e) URO; (f) ZNFP. Ticks on branches indicate mutational steps between haplotypes. Circle size is proportional to the number of individuals having a haplotype.

To better explore this unexpected pattern, we constructed haplotype networks of the gene fragments studied with *R*. *neglectus*, *R*. *taquarussuensis* and *R*. *prolixus* (Fig 2). In the case of CYTB, we found *R*. *prolixus* separated from the other two species by 15 mutational steps. In contrast, *R*. *taquarussuensis* haplotypes were less distant to *R*. *neglectus* (only two mutational steps). In fact, the divergence of *R*. *taquarussuensis* from *R*. *neglectus* (H.1 and H.2) is less than the divergence between such haplotypes and others from the same species (i.e. H.3 to H.8). Consistently, nucleotide diversity of *R*. *prolixus* and *R*. *neglectus* is higher than that of *R*. *taquarussuensis* (Table 3).

**Fig 3:**
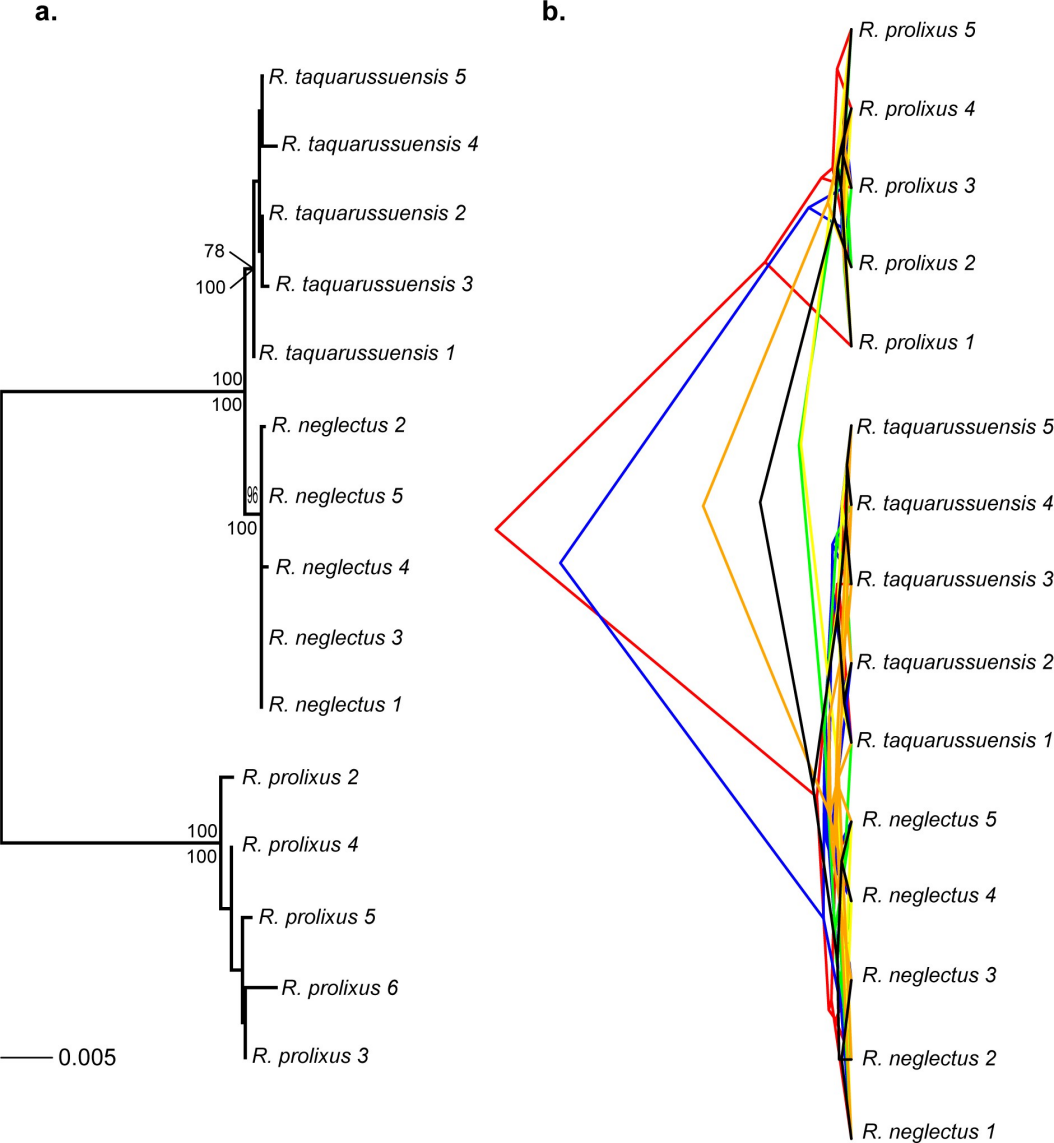
Phylogenetic trees for *R*. *prolixus*, *R*. *neglectus* and *R*. *taquarussuensis* based on all molecular markers. A. Multilocus phylogeny where node support is indicated on each branch: bootstrap (above) and posterior probability (below). B. Bayesian superimposed gene trees: red (CYTB), blue (ND4), green (TOPO), yellow (URO), orange (PCB) and black (ZNFP). The alignment consisted of 3731 bp.

**Fig 4:**
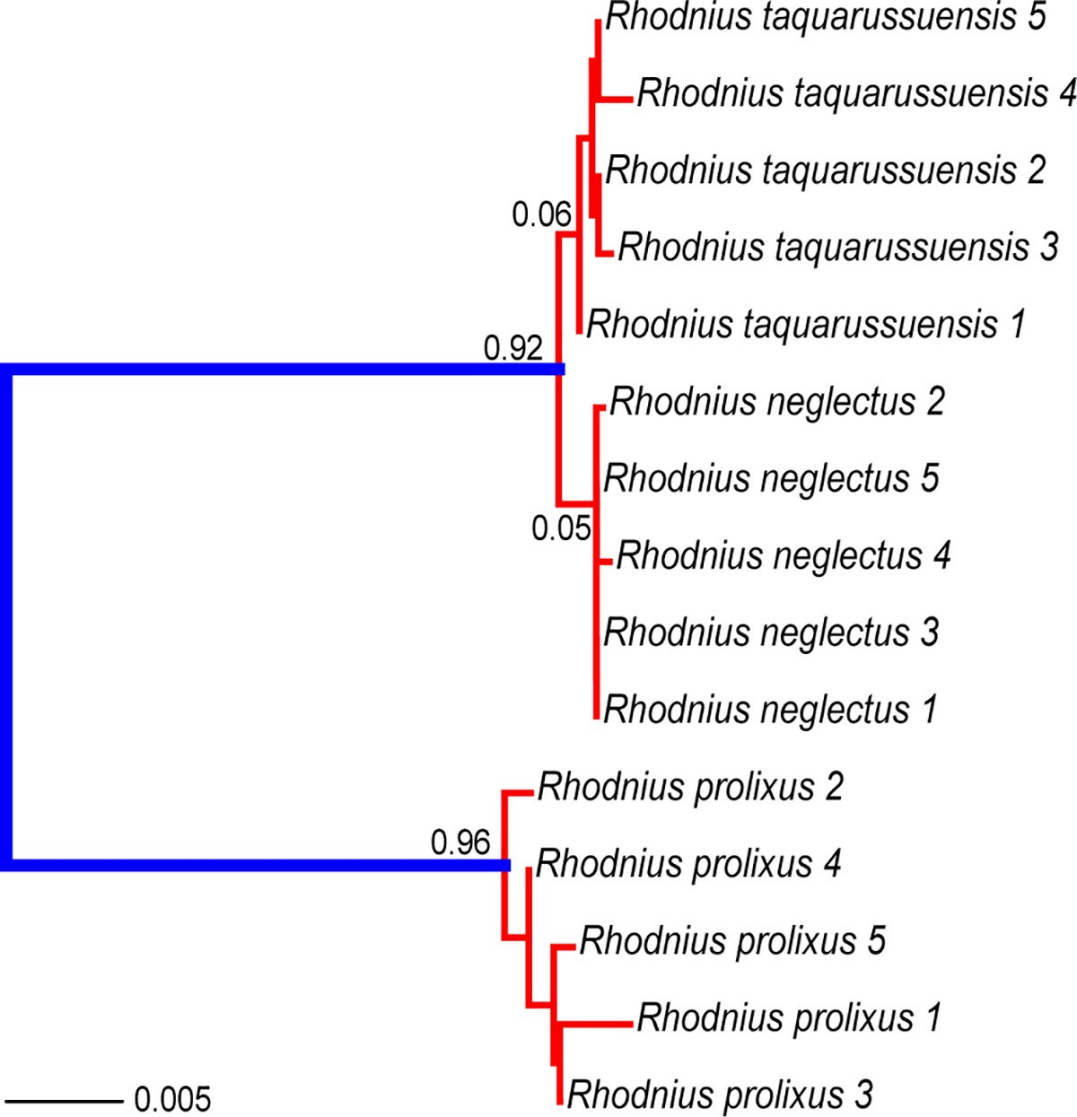
Species delimitation based on the Poisson Tree Process (PTP). Maximum Likelihood and Bayesian inference yielded identical results. Numbers on each node are posterior probabilities of the inner taxa forming one species. Thus, red branches indicate taxa that should be considered as part of the same lineage.

We recovered the same multilocus phylogeny for *R*. *prolixus*, *R*. *neglectus* and *R*. *taquarussuensis* with ML and Bayesian approaches (ML substitution models were CYTB: HKY+F+I; ND4: HKY+F; PCB: F81+I; TOPO: F81+I; URO: HKY+F; ZNFP: TPM2+F+I). The three species were monophyletic and all of them with posterior probabilities of 100 (Fig 3A) Bootstrap support values were > 90 for *R*. *prolixus* and *R*. *neglectus*, while *R*. *taquarussuensis* has a bootstrap support of 78. Also, the branch length of *R*. *taquarussuensis* is less than one in a thousand changes. The unlinked and superimposed Bayesian gene trees consistently recovered two main clades: one exclusively composed of *R*. *prolixus*, and the second where *R*. *neglectus* and *R*. *taquarussuensis* show incomplete coalescence (Fig 3B). Consistently, in the analysis of species delimitation (PTP), both the Maximum Likelihood and Bayesian inference found two species as the most probable partition (Fig 4). These two partitions correspond to *R*. *prolixus* and *R*. *neglectus*. All other internal nodes had probabilities lower than 0.1 (Fig 4).

### Genetic differentiation

Overall, all markers showed low genetic diversity for the three taxa, *R*. *prolixus*, *R*. *neglectus* and *R*. *taquarussuensis*. In particular, the loci PCB and ND4 showed the same pattern as CYTB, where *R*. *taquarussuensis* is less diverse than the other two species (Table 4). The remaining loci showed *R*. *taquarussuensis* less diverse than *R*. *prolixus* and the diversity of *R*. *neglectus* was zero. This is consistent with the low number of haplotypes observed in the haplotype networks (Fig 2), where *R*. *prolixus* has private haplotypes that clearly differentiate it from the other two species (Fig 2B–2F), while *R*. *taquarussuensis* and *R*. *neglectus* exhibit substantial haplotype sharing (Fig 2).

**Table 4:** Absolute genetic divergence corrected by nucleotide diversity (Da) and Kimura 2 Parameter distance (K2P) between *R*. *prolixus*, *R*. *taquarussuensis* and *R*. *neglectus*.

Gene	Species pair	Da	K2P
CYTB	*R*. *neglectus–R*. *taquarussuensis*	0.003	0.003
	*R*. *neglectus–R*. *prolixus*	0.06639	0.082
	*R*. *taquarussuensis–R*. *prolixus*	0.06939	0.086
ND4	*R*. *neglectus–R*. *taquarussuensis*	0	0
	*R*. *neglectus–R*. *prolixus*	0.0625	0.075
	*R*. *taquarussuensis–R*. *prolixus*	0.0625	0.075
PCB	*R*. *neglectus–R*. *taquarussuensis*	0.00037	0.001
	*R*. *neglectus–R*. *prolixus*	0.0359	0.038
	*R*. *taquarussuensis–R*.*prolixus*	0.0359	0.039
TOPO	*R*. *neglectus–R*. *taquarussuensis*	0.00221	0.004
	*R*. *neglectus–R*. *prolixus*	0.01325	0.014
	*R*.*taquarussuensis–R*. *prolixus*	0.01545	0.018
URO	*R*. *neglectus–R*. *taquarussuensis*	0.00476	0.005
	*R*. *neglectus–R*. *prolixus*	0.01701	0.017
	*R*. *taquarussuensis–R*. *prolixus*	0.01701	0.012
ZNFP	*R*. *neglectus–R*. *taquarussuensis*	0.00635	0.007
	*R*. *neglectus–R*. *prolixus*	0.02234	0.024
	*R*. *taquarussuensis–R*. *prolixus*	0.02585	0.028

Consistent with these findings, D_XY_ shows *R*. *prolixus* highly differentiated from *R*. *neglectus* and *R*. *taquarussuensis* in all loci whilst the latter two taxa do not differentiate between them (S2 Fig). When correcting for the nucleotide diversity, the same pattern is observed (Table 4). The genetic distance (K2P) between *R*. *neglectus* and *R*. *taquarussuensis* in all loci was less than 7.5%, a value previously used to define species in triatomines using CYTB [43]. Also, the discriminant analysis = of genetic variation for both mtDNA and nDNA fails to separate the taxa *R*. *neglectus* and *R*. *taquarussuensis*, which is reflected by the overlap of their densities on the canonical function (S3 Fig).

### Interspecific crosses

All interspecific matings attempted were successful (n = 6), suggesting that there are no mechanical and/or gametic mechanisms that act against hybridization between *R*. *neglectus* and *R*. *taquarussuensis*. When we tested homogeneity across categories in the hatching rate, we did not observe differences between interspecific crosses (direct or reciprocal) and controls (Table 5; G_6_ = 7.06, *P* = 0.3152). Models that have multiple means (G_3_ = 1.243, *P* = 0.7428) or variances (G_3_ = 2.097, *P* = 0.5525) for the hatching rate were not supported by the data, indicating the absence of maternal or cytoplasmic effects.

**Table 5:** Results for interspecific and conspecific crosses. R denotates replicate number for each cross. SE = standard error.

Type of cross		Laid eggs (hatched)				Proportion of viable eggs (SE)	Variance (SE)
		R1	R2	R3	Total		
Interspecific	*R*. *taquarussuensis* ♀ x *R*. *neglectus* ♂	230 (198)	86 (80)	230 (193)	510 (471)	0.83 (0.03)	0.0016 (0.002)
	*R*. *neglectus* ♀ x *R*. *taquarussuensis* ♂	300 (275)	181 (105)	256 (244)	708 (624)	0.88 (0.02)	0.0006 (0.0007)
Conspecific	*R*. *neglectus* ♀ x *R*. *neglectus* ♂	337 (308)	409 (346)	174 (155)	901 (809)	0.86 (0.02)	0.0001 (0.0016)
	*R*. *taquarussuensis* ♀ x *R*. *taquarussuensis* ♂	151 (127)	168 (150)	201 (156)	501 (433)	0.78 (0.14)	0.034 (0.046)

## Discussion

*Rhodnius* exhibits morphological traits that facilitate its identification at the genus level [18, 51], but the low morphological variation within the genus precludes an easy species identification based on morphology alone [23]. This has led to suggest the existence of cryptic species in *Rhodnius*, where multiple look-alike lineages should be considered as different species based on their genetic differentiation [11, 16, 23, 51]. However, morphological species identification in *Rhodnius* relies on intraspecifically variable traits, which can lead to over-estimate the number of species [5]. Therefore, it is necessary to validate the status of the currently described species in the genus implementing a comprehensive approach that uses morphology, genetics, and measures of reproductive isolation.

*R*. *taquarussuensis* is the most recently described species in *Rhodnius*, based on morphological, morphometric and cytogenetic evidence [22]. However, the description of this species lacked other crucial evidence. Here, we tested the species status of *R*. *taquarussuensis* sequencing six molecular markers and performing interspecific crosses. Our results suggest that, despite the morphological differences between *R*. *taquarussuensis* and *R*. *neglectus* [22], these taxa constitute a single species.

**Fig 5:**
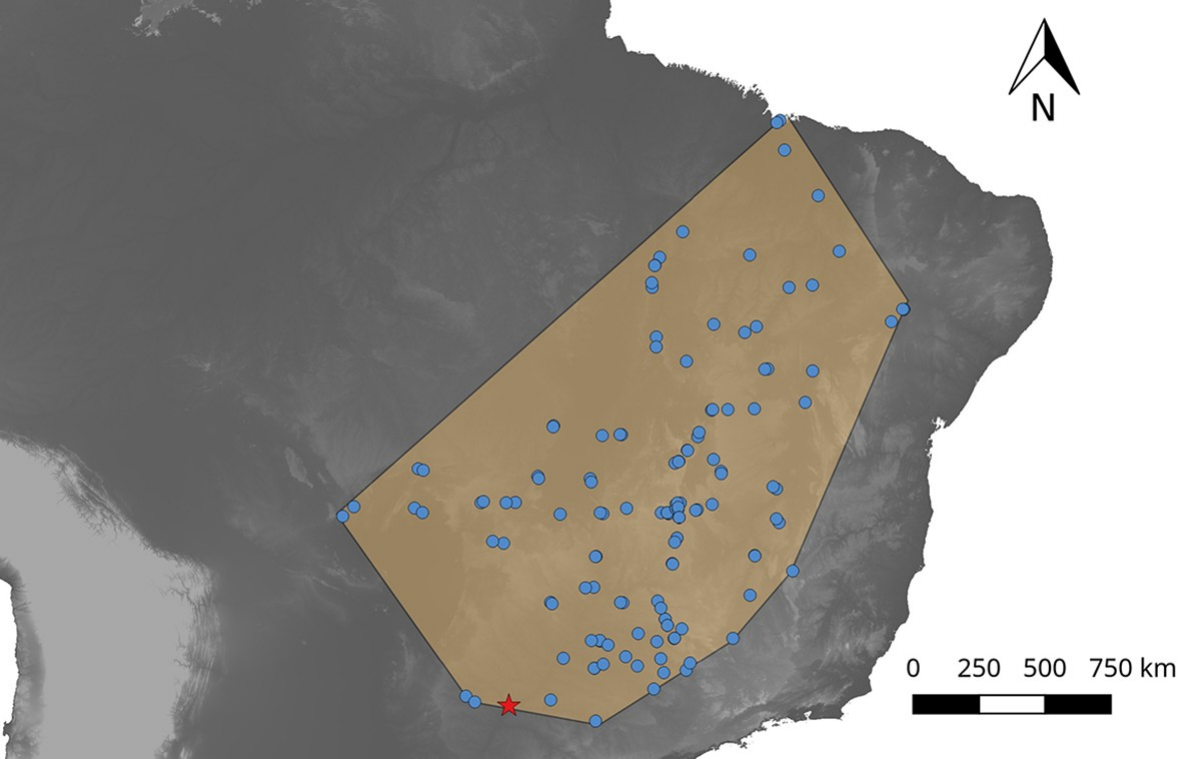
Geographical distribution of *R*. *neglectus* (blue) and *R*. *taquarussuensis* (red). Distribution of *R*. *neglectus* is based on records available on DataTri [55] whilst that of *R*. *taquarussuensis* is based on collections made by the authors.

Firstly, the known distribution range of *R*. *taquarussuensis* overlaps that of *R*. *neglectus* (Fig 5). Thus, for them to be different species it would be necessary to evolve strong intrinsic and/or extrinsic isolation barriers that restrict gene flow. In contrast, we found that *R*. *taquarussuensis* and *R*. *neglectus* successfully cross and there are no maternal or cytoplasmic effects that affect offspring viability, as reflected by the high hatching rates we obtained. This also suggests the absence of mechanical or gametic mechanisms acting against their hybridization. Although we did not test the fertility of the “hybrid” offspring, the egg viability observed in our crosses is higher than that reported for other interspecific crosses between different species in the subfamily Triatominae, where hybrid disfunction has been detected [47, 52–54]. However, the role of other pre-zygotic barriers such as temporal asynchrony, mate choice and/or habitat differences, among others, remains to be tested.

Secondly, our phylogenies and haplotype networks showed *R*. *taquarussuensis* nested within *R*. *neglectus*, with no differentiation from this species. Consequently, the species delimitation analysis collapsed these two taxa as a single one. Additionally, genetic differentiation measures as well as the discriminant analysis failed to show genetic structure between these lineages. Recent genomic analysis in animals have established that ‘good-species’ usually have a genetic divergence (Da) > 2%, although there is a “grey zone” of speciation (in which taxonomy is often controversial), that spans from 0.5% to 2% of Da. However, any Da < 0.5% undoubtedly corresponds to populations of the same species [56]. Therefore, our Da values are consistent with a scenario of *R*. *taquarussuensis* being *R*. *neglectus* rather than a different species. Furthermore, our genetic distance (K2P) estimates between *R*. *neglectus* and *R*. *taquarussuensis* were lower than those between *R*. *neglectus* and *R*. *prolixus*, and between *R*. *taquarussuensis* and *R*. *prolixus*. This genetic similarity between *R*. *taquarussuensis* and *R*. *neglectus* in all our analyses contrast with the clear differentiation observed between *R*. *neglectus* and *R*. *prolixus*, which are known to be distinct yet closely related species. In agreement with these findings, recent studies have suggested that *R*. *milesi* (Carcavalho et al., 2001), another species described based on cytogenetic differences [57, 58], shows high genetic similarity with *R*. *neglectus* thus questioning its validity as a true species [11]. This further suggests that *R*. *neglectus* may be a species that shows important polymorphism in cytogenetic patterns, which should not be used for species diagnosis.

The original description of *R*. *taquarussuensis* reported differences in the constitutive heterochromatin pattern and nanocomposition of TA and CG rich DNA base pairs between *R*. *taquarussuensis* and *R*. *neglectus*, mainly because *R*. *taquarussuensis* shows more heterochromatic blocks in the autosomes and the Y chromosome compared to the other *Rhodnius* species. Although gain and/or loss of constitutive heterochromatin has been previously used as evidence of species differentiation in the *R*. *pallescens* group [59], the *T*. *sordida* subcomplex [60, 61], and *T*. *dimidiata* (Latreille, 1811) [62], such heterochromatin differences between *R*. *neglectus* and *R*. *taquarussuensis* are likely just intraspecific polymorphism of *R*. *neglectus*. The presence of intraspecific heterochromatin variation with no apparent consequences on speciation is not new in Triatominae and has been observed in *T*. *infestans* (Klug, 1834) [63–65], *P*. *geniculatus* (Latreille, 1811) [66], and *R*. *pallescens* [67]. Therefore, although cytogenetics is a valuable methodology for taxonomic studies [68], heterochromatin variation between populations (i.e. the existence of cytotypes) is not a reliable trait to delimit species when evaluated alone. This agrees with the fact that cytogenetics is known to have a 20% failure rate in delimiting arthropods’ species [69]. In conclusion, after performing a comprehensive analysis using mitochondrial and newly developed nuclear markers, as well as crosses between *R*. *taquarussuensis* and *R*. *neglectus*, we can confidently suggest that *R*. *taquarussuensis* is not a separate species and must be considered a synonym of *R*. *neglectus*. Our study highlights the importance of revising carefully the current taxonomy of *Rhodnius*, because only a confident species delimitation will permit to study the processes and mechanisms involved in their diversification, as well as to unveil vector/parasite associations with epidemiological relevance.

## Supporting information

S1 Table CYTB accession number for individuals downloaded from GenBank. (DOCX)

S1 Fig CYTB Maximum likelihood phylogeny. (DOCX)

S2 Fig Absolute genetic divergence (DXY) between *R*. *prolixus*, *R*. *neglectus* and *R*. *taquarussuensis*. (a) CYTB; (b) ND4; (c) PCB; (d) TOPO; (e) URO; (f) ZNFP. Note that DXY scale for all genes is not the same.
(DOCX)

S3 Fig Discriminant analysis based on mtDNA (a) and nDNA (b). Densities for a single discriminant function are shown, with red being *R*. *taquarussuensis* and blue being *R*. *neglectus*.
(DOCX)
