# Correction: Taxonomical over splitting in the *Rhodnius prolixus* (Insecta: Hemiptera: Reduviidae) clade: Are *R*. *taquarussuensis* (da Rosa et al., 2017) and *R*. *neglectus* (Lent, 1954) the same species?

**DOI:** 10.1371/journal.pone.0213043

**Published:** 2019-02-22

**Authors:** The PLOS ONE Staff

There are errors in the Funding statement. The correct Funding statement is as follows: This work was funded by DIRECCION DE INVESTIGACION E INNOVACION from UNIVERSIDAD DEL ROSARIO "Big grant: Genómica, evolución y biogeografía de especies del género *Rhodnius*: vectores de la enfermedad de Chagas—IV-FGD002". CS, CP and FS were funded by DIRECCION DE INVESTIGACION E INNOVACION FONDOS CONCURSABLES 2018, Universidad del Rosario—MODALIDAD SEMILLEROS-IV-ACA008. The funders had no role in study design, data collection and analysis, decision to publish, or preparation of the manuscript. The publisher apologizes for the error.

## References

[1] Nascimento JD, da Rosa JA, Salgado-Roa FC, Hernández C, Pardo-Diaz C, Alevi KCC, et al (2019) Taxonomical over splitting in the *Rhodnius prolixus* (Insecta: Hemiptera: Reduviidae) clade: Are *R*. *taquarussuensis* (da Rosa et al., 2017) and *R*. *neglectus* (Lent, 1954) the same species? PLoS ONE 14(2): e0211285 10.1371/journal.pone.0211285 30730919 PMC6366742

