# Supplementary material for: Taxonomical over splitting in the Rhodnius prolixus (Insecta: Hemiptera: Reduviidae) clade: Are R. taquarussuensis (da Rosa et al., 2017) and R. neglectus (Lent, 1954) the same species?
Source: PLoS One. 2019 Feb 7;14(2):e0211285. doi: 10.1371/journal.pone.0211285 (PMC6366742; doi:10.1371/journal.pone.0211285)
Supplement: S1 Table — (DOCX) [file pone.0211285.s001.docx]

S1 Table. CYTB accession number for individuals downloaded from GenBank

| **Species** | **Accession number** |
| --- | --- |
| *Rhodnius montenegrensis* | KR072682.1 |
| *Rhodnius nasutus* | JX273155.1 |
| *Rhodnius neglectus* | AF045716.1 |
|  | JX273156.1 |
|  | KT317034.1 |
|  | KT317035.1 |
|  | KT317036.1 |
|  | KT317037.1 |
|  | KT317038.1 |
|  | KT317039.1 |
|  | KT317040.1 |
|  | KT317041.1 |
|  | KT317042.1 |
|  | KT317043.1 |
|  | KT317044.1 |
|  | KT317045.1 |
|  | KT317046.1 |
|  | KT317047.1 |
|  | KT317048.1 |
|  | KT317049.1 |
|  | KT317050.1 |
|  | KT317051.1 |
|  | KT317052.1 |
|  | KT317053.1 |
|  | KT317054.1 |
|  | KT317055.1 |
|  | KT317056.1 |
|  | KT317057.1 |
|  | KT317058.1 |
|  | KT317059.1 |
|  | KT317060.1 |
|  | KT317061.1 |
|  | KT317062.1 |
|  | KT317063.1 |
|  | KT317064.1 |
|  | KT317065.1 |
|  | KT317066.1 |
|  | KT317067.1 |
|  | KT317068.1 |
| *Rhodnius prolixus* | AF045718.1 |
|  | AF421339.1 |
|  | EF011716.1 |
|  | EF011721.1 |
|  | EF011723.1 |
|  | EF011726.1 |
|  | EF043576.1 |
|  | EF043577.1 |
|  | EF043578.1 |
|  | EF043579.1 |
|  | EF043580.1 |
|  | EF043581.1 |
|  | EF043582.1 |
|  | EF043583.1 |
|  | EF043584.1 |
|  | EF043585.1 |
|  | EF043586.1 |
|  | EF043587.1 |
|  | EF043588.1 |
|  | KC543514.1 |
|  | KP126725.1 |
|  | KP126726.1 |
|  | KP126727.1 |
|  | KP126728.1 |
|  | KP126729.1 |
|  | KP126730.1 |
|  | KP126731.1 |
|  | KP126732.1 |
|  | KP126733.1 |
|  | KP126734.1 |
| *Rhodnius robustus* | AF045717.1 |
|  | AF421340.1 |
|  | AF421341.1 |
|  | AF421342.1 |
|  | AF421343.1 |
|  | EF011708.1 |
|  | EF011709.1 |
|  | EF011710.1 |
|  | EF011711.1 |
|  | EF011712.1 |
|  | EF011713.1 |
|  | EF011714.1 |
|  | EF011715.1 |
|  | EF011717.1 |
|  | EF011718.1 |
|  | EF011719.1 |
|  | EF011720.1 |
|  | EF011722.1 |
|  | EF011724.1 |
|  | EF011725.1 |
|  | EF011727.1 |
|  | EF011728.1 |
|  | EF071583.1 |
|  | FJ887793.1 |
|  | JN831953.1 |
|  | JN831954.1 |
|  | JN831955.1 |
|  | JN831956.1 |
|  | JN831957.1 |
|  | JN831958.1 |
|  | JN831959.1 |
|  | JN831960.1 |
|  | JN831961.1 |
|  | JN831962.1 |
|  | JN831963.1 |
|  | JN831964.1 |
|  | JN831965.1 |
|  | JN831966.1 |
|  | JN831967.1 |
|  | JN831968.1 |
|  | JN831969.1 |
|  | JN831970.1 |
|  | JX273158.1 |
|  | KT805149.1 |
|  | MF966277.1 |
|  | MF966278.1 |
|  | MF966279.1 |
|  | MF966280.1 |
|  | MF966281.1 |
|  | MF966282.1 |
|  | MF966283.1 |
|  | MF966284.1 |
|  | MF966285.1 |
|  | MF966286.1 |
|  | MF966287.1 |
|  | MF966288.1 |
|  | MF966289.1 |
|  | MF966290.1 |
|  | MF966291.1 |
|  | MF966292.1 |
|  | MF966293.1 |
|  | MF966294.1 |
|  | MF966295.1 |
|  | MF966296.1 |
|  | MF966297.1 |
|  | MF966298.1 |
|  | MF966299.1 |
|  | MF966300.1 |
|  | MF966301.1 |
|  | MF966302.1 |
|  | MF966303.1 |
|  | MF966304.1 |
|  | MF966305.1 |
|  | MF966306.1 |
| *Rhodnius barreti* | JX273159.1 |
|  | JX273160.1 |
|  | JX273161.1 |
|  | JX273162.1 |
