# Supplementary material for: Taxonomical over splitting in the Rhodnius prolixus (Insecta: Hemiptera: Reduviidae) clade: Are R. taquarussuensis (da Rosa et al., 2017) and R. neglectus (Lent, 1954) the same species?
Source: PLoS One. 2019 Feb 7;14(2):e0211285. doi: 10.1371/journal.pone.0211285 (PMC6366742; doi:10.1371/journal.pone.0211285)
Supplement: S1 Fig — (DOCX) [file pone.0211285.s002.docx]

S1 Figure. Cytb Maximum likelihood phylogeny.
