# Supplementary material for: Taxonomical over splitting in the Rhodnius prolixus (Insecta: Hemiptera: Reduviidae) clade: Are R. taquarussuensis (da Rosa et al., 2017) and R. neglectus (Lent, 1954) the same species?
Source: PLoS One. 2019 Feb 7;14(2):e0211285. doi: 10.1371/journal.pone.0211285 (PMC6366742; doi:10.1371/journal.pone.0211285)
Supplement: S2 Fig — (a) CYTB; (b) ND4; (c) PCB; (d) TOPO; (e) URO; (f) ZNFP. Note that DXY scale for all genes is not the same. (DOCX) [file pone.0211285.s003.docx]

**S2 Fig. Absolute genetic divergence (D_XY_) between *R. prolixus, R. neglectus* and *R. taquarussuensis.*** (a) Cytb; (b) ND4; (c) PCB; (d) TOPO; (e) URO; (f) ZNFP. Note that D_XY_ scale for all genes is not the same.
