# Supplementary material for: Taxonomical over splitting in the Rhodnius prolixus (Insecta: Hemiptera: Reduviidae) clade: Are R. taquarussuensis (da Rosa et al., 2017) and R. neglectus (Lent, 1954) the same species?
Source: PLoS One. 2019 Feb 7;14(2):e0211285. doi: 10.1371/journal.pone.0211285 (PMC6366742; doi:10.1371/journal.pone.0211285)
Supplement: S3 Fig — Discriminant analysis based on mtDNA (a) and nDNA (b). Densities for a single discriminant function are shown, with red being R. taquarussuensis and blue being R. neglectus. (DOCX) [file pone.0211285.s004.docx]

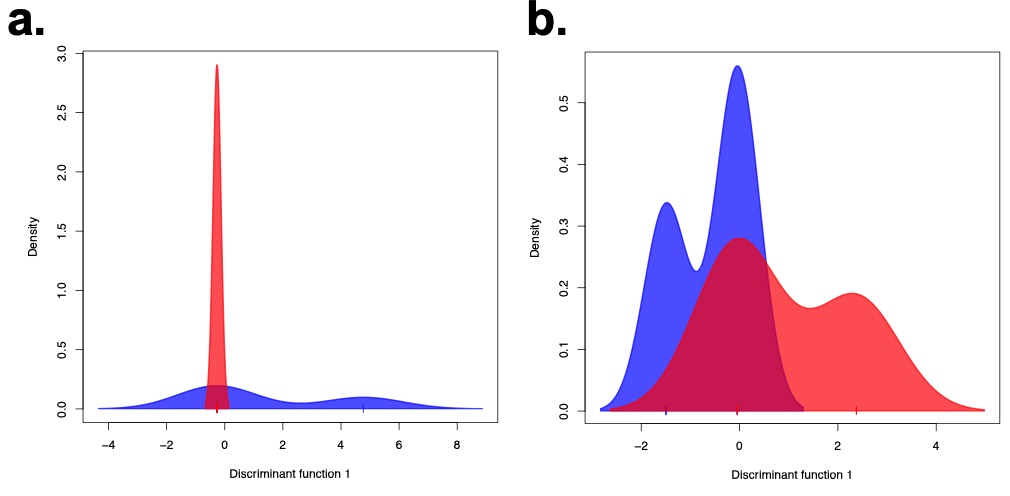


**S3 Fig. Discriminant analysis based on mtDNA (a) and nDNA (b).** Densities for a single discriminant function are shown, with red being *R. taquarussuensis* and blue being *R. neglectus.*
